# News and Coming Events

**Published:** 1907-07-13

**Authors:** 


### NEWS AND COMING EVENTS.

The Late Sir William Broadbent.—The announcement of the death of Sir William Broadbent, physician-in-ordinary to H.M. the King, which occurred at his residence, 84 Brook Street, Grosvenor Square, W., early on Wednesday morning, the 10th instant, will be received with keen regret. In medicine Sir William Broadbent's name has always occupied a high position, and his personal efforts towards the furtherance of medical science have received appreciative recognition both at home and abroad. The Universities of Edinburgh, St. Andrews, and Leeds conferred on him honorary degrees of LL.D. and D.Sc.; he was a corresponding member of many learned French and German societies; a Fellow of the Royal Society since 1884; President of the British Medical Benevolent Fund; Chairman of Council of the National Association for the Prevention of Tuberculosis; Vice-President of the Cancer Research Fund; President of the Harveian, Medical, Clinical, and Neurological Societies; and for two terms filled the post of Censor to the Royal College. He took an active and keen interest in French medicine, and was Commander of the Legion of Honour. Born in 1835, he received his medical education at Manchester, in Paris, and in London, and soon after qualifying in 1858 was appointed to the medical staff of St. Mary's Hospital, which institution he served up to the time of his death both actively and as a consultant. As a teacher his claims to remembrance are too well known to the many who have had the privilege of listening to his lectures or studying under him in any other capacity to need more than passing mention. In 1898 he was appointed physician-extraordinary to the late Queen Victoria, and physician-in-ordinary to H.R.H. the Prince of Wales. He was created a baronet in 1893, and later on was made a K.C.V.O.

An extraordinary general meeting of the Hampstead General Hospital will be held at the hospital, Haverstock Hill, on Tuesday, July 16, at 6 p.m., when the council will lay before the members of the Institute the arrangements which have been made for amalgamation with the North-West London Hospital. The following resolutions will be submitted in pursuance of a notice received—namely: 1. That the practice hitherto followed of appointing acting medical officers from among the members of the medical profession practising in Hampstead be continued. 2. That the number of physicians and surgeons on the consulting staff be augmented.

The third annual general meeting of the Association of Scottish Medical Diplomates was held on June 19, at 11 Chandos Street, London, W. The report of the Council for the past year disclosed a flourishing condition of affairs. It was unanimously adopted by the meeting, together with the Treasurer's balance-sheet and a draft copy of rules, submitted by the Council. The retiring President, Dr. A. Farrer, was elected an honorary vice-president of the Association. Dr. David Walsh was unanimously appointed to the office of President for the ensuing year, 1907-1908, while Mr. Sydney Stephenson, F.R.C.S., was re-elected Treasurer, and Dr. Arthur Harries Honorary Secretary for a corresponding period. Dr. Skene Reeth and Mr. Charles Ryall were appointed to vacancies on the Executive Council. The new President congratulated the members present on the prosperity of the Association, for which he anticipated a brilliant future. Scottish diplomates desirous of obtaining further information as to the objects of the Association are requested to write to the Hon. Secretary, 11 Chandos Street, London, W.

The Countess of Ilchester has consented to accept the Presidency of the Ladies' Committee of the Chelsea Hospital for Women.

Among the natural history exhibits at the forthcoming Alaska Yukon-Pacific Exposition, to be held at Seattle in 1909, will be a perfectly preserved mammoth. The carcass of the animal was discovered in an Alaskan ice formation, and is now being preserved in a refrigerating chamber.

A special appeal for £70,000 is being made for a comprehensive scheme of extension at the Salford Royal Hospital. The Board of Management has decided to increase the number of beds so as to provide accommodation for two hundred in-patients, and also to make suitable provision for the nursing staff.

The new electrical department of the Royal Devon and Exeter Hospital, Exeter, will be opened on Friday, July 19, next, at 4 p.m., by Lady Duckworth-King. The building and fittings are the gift of Mrs. Sanders in memory of her husband and son, formerly Presidents of the hospital.

A conversazione was held on July 3 in the buildings and grounds of the Cancer Hospital, Fulham Road, Lord Ludlow, president of the hospital, accompanied by Lady Ludlow, received the guests, among whom were a large number of medical practitioners. The medical and surgical staff and other officers of the hospital assisted the president in conducting the guests through the wards and the various departments, where the facilities both for treatment and research were fully explained. There are now 114 beds available, and, as the hospital is quite free, letters of recommendation are not required. In the wards every form of treatment which appears to offer any possible hope of relief is given an exhaustive trial. The electrical department, the pathological and research departments, the new museum and the chapel, which is in telephonic communication with every bed, were all open for inspection, and the string band of the Coldstream Guards played in the grounds. An appeal is being made for funds to meet the increasing expenditure of the hospital, which greatly exceeds the ordinary income. Last year the ordinary income amounted to £9,013, and the expenditure to £16,699, making a deficit of £7,686.

Metropolitan Hospital Sunday Fund.—The following are among the amounts received at the Mansion House to date (July 5):—Holy Trinity, Sloane Street, £414; St. Jude, South Kensington, £360; St. George, Hanover Square, with St. Mary, Bourdon Street, £301; St. Columba, Pont Street, Church of Scotland, £195; St. Peter, Brockley, £109; Holy Trinity, St. Marylebone, £79; Stamford Hill Congregational Church, £70; St. Luke, Chelsea, £70; Victoria Park Christian Evidence Association, £67; St. Mary, Hornsey Rise, £61; Rosslyn Hill Unitarian Church, £59; Christ Church, Crouch End, £57; Christ Church, Hampstead, and Mission, £53; Barking Parish Church and Chapels, £51; Newington Parish Church and Chapel-of-Ease, £41; St. Stephen and Church of Transfiguration, Lewisham, £36; Trinity Presbyterian Church, Notting Hill, £32; Greenwich Parish Church and Chapel-of-Ease, £32; St. James, Notting Hill, £31; St. Mary Magdalene, Peckham, £31; St. Mary, Walthamstow, £29; Christ Church, Southgate, £27; St. Mark, Notting Hill, £25; All Saints, North Peckham, £25; St. Olave, Hart Street, £23; Charterhouse Chapel, £23; St. Paul, Mill Hill, £22; Lewisham High Road Congregational Church, £22; St. Helen, North Kensington, £20. Total, about £40,000.

